# Tears for pears: Influence of children’s neophobia on categorization performance and strategy in the food domain

**DOI:** 10.3389/fnut.2022.951890

**Published:** 2022-09-21

**Authors:** Damien Foinant, Jérémie Lafraire, Jean-Pierre Thibaut

**Affiliations:** ^1^LEAD – CNRS UMR-5022, Université Bourgogne Franche-Comté, Dijon, France; ^2^Institut Paul Bocuse Research Center, Écully, France

**Keywords:** food neophobia, categorization, signal detection theory, food processing, children

## Abstract

Preschoolers’ neophobic dispositions mainly target fruits and vegetables. They received a great deal of attention in the past decades as these dispositions represent the main psychological barrier to dietary variety. Recently, children’s food neophobia has been found to be negatively correlated with their categorization performance (i.e., the accuracy to discriminate between food categories). We investigated categorization strategies among neophobic children, tendencies to favor one type of error over the other (misses over false alarms), in order to compensate for their poor categorization performance. To capture children’s categorization strategies, we used the Signal Detection Theory framework. A first experiment assessed 120 3-to-6-years old children’ sensitivity to discriminate between foods and nonfoods as well as their decision criterion (i.e., response strategy). In a second experiment, we manipulated the influence of food processing. The hypothesis was that food processing acts as a sign of human interventions that decreases uncertainty about edibility and thus promotes feelings of safety in the food domain. 137 children were tested on a food versus nonfood categorization task contrasting whole and sliced stimuli. In both experiments, increased levels of food neophobia were significantly associated with poorer categorization sensitivity and with a more conservative decision criterion (i.e., favoring “it is inedible” errors). Additionally, results from Experiment 2 revealed that food processing did not influence neophobic children, whereas their neophilic counterparts adopted a more liberal decision criterion for sliced stimuli than for whole stimuli. These findings are the first demonstration of a relationship between a decision criterion and food neophobia in young children. These results have strong implications for theories of food neophobia and laid the groundwork for designing novel types of food education interventions.

## Introduction

Why avoid patting an animal we see for the first time? Why not pick up an unfamiliar fruit from its tree to stave our hunger? Fearful reactions toward unfamiliar stimuli or situations are referred to as *neophobia* (1–3). Neophobia is a widespread disposition in human and non-human animals, on a continuum from less neophobic (or even neophilic) to neophobic individuals (4). On the neophobic endpoint, individuals show aversive reactions (e.g., avoidance) toward every stimulus or situation they are uncertain about. On the opposite endpoint also called neophilia, individuals are attracted by novelty (4). They tend to actively seek new sensations (5) and are open to novel experiences (6).

A great deal of interest in neophobia comes from its manifestation in the food domain, especially in human children [see refs. (7, 8) for reviews]. This is because high levels of food neophobia can have negative consequences for healthy development by hindering dietary variety, particularly the consumption of fruits and vegetables (9). Related to food neophobia, yet distinct, food pickiness is another barrier to children’s dietary variety, defined as the rejection of a substantial number of familiar, including already tasted, foods (10). Controversy exists concerning their relationship. For some authors, food neophobia is a subset of pickiness [e.g., ref. (7)], whereas others claim that they are distinct theoretically and behaviorally (4, 11, 12) as they have different predictors. Although food neophobia and pickiness have an increased prevalence during childhood, such dietary habits and behaviors prevail well into adulthood (13). It is therefore critical to understand the cognitive underpinnings of food neophobia and picky/fussy eating as well as the factors that could contribute to mitigating these two types of food rejection.

Recent studies have evidenced that the intensity of food neophobia was negatively related to children’s performance in food categorization and induction tasks [e.g., ref. (14)]. Although much research has examined children’s ability to discriminate categories of items in the food domain, less is known about their categorization strategies. In an uncertain situation (e.g., when the food is novel or when discrimination is difficult), knowing that errors differ in their consequences (for instance deciding whether black, small, and juicy-looking berries are edible), there are two possible strategies. The first, conservative, strategy is to exercise caution as these berries are difficult to identify and could be toxic. The second, liberal strategy is more daring and consists in accepting the berries as edible, despite the uncertainty. Both strategies have advantages and disadvantages. Being conservative avoids dangers, choking, poisoning, death. However, this strategy can deprive individuals of a nutrient source but also of the opportunity to expand their knowledge of new foods. By being liberal, an individual accepts the risks associated with uncertainty but benefits from the opportunity to expand both their food repertoire and their category of edible items. The present study compares both the categorization performance and strategies of neophobic and neophilic children. In what follows, we start with a summary regarding food neophobia and the differences between neophobic and neophilic children. Then, we review more specifically the association between food neophobia and categorization.

Food neophobia is generally observed during early childhood (between 2 and 6 years). It refers to the tendency to reject novel or unknown foods at mere sight (15). This rejection occurs before the food is tasted and is thought to have an evolutionary protective function for children, minimizing the risk of ingesting novel and potentially harmful items (16). However, severe food neophobia has been linked to poor dietary habits such as a reduced dietary variety and lower consumption of vegetables (7, 11, 17). Numerous studies have shown that the intensity of food neophobia is stable between 2 and 6 years (12, 18–22). For instance, Kozioł-Kozakowska et al. (21) tested whether the proportions of children scoring “low,” “average,” and “high” on a food neophobia scale varied across age groups between 2 and 7 years. Their results showed that the majority of children, almost 80%, was scoring in the middle of the scale. The 20% of children left were equally divided into neophilic (i.e., low neophobia) and highly neophobic. Importantly, the authors did not find any significant difference in the proportion of these three groups when they compared the youngest children and the oldest children. Moreover, the impact of a child’s food neophobia extends beyond childhood, since dietary habits acquired during this period partly determine dietary patterns in adulthood (13). Considering the importance of dietary variety across the lifespan, researchers have focused on understanding the mechanisms underpinning food neophobia in young children.

Since eating is socially grounded, social and environmental factors are important during the period of food neophobia. The caregiver’s characteristics significantly affect children’s food neophobia. For instance, children’s food neophobia has been found to be positively correlated with parental food neophobia (12, 23) and negatively correlated with socioeconomic status (24–26) and educational level (27). Parental feeding practices are also important in weakening or strengthening children’s food neophobia (28). For example, common parental feeding strategies such as food rewards, or pressure to eat, increase children’s food neophobia tendencies (29). In contrast, introducing a high variety of vegetables at weaning has a positive impact (30). Another major influence of the social context on children’s reaction to food is social facilitation (31), defined as an increase in the probability of performing a class of behavior in the presence of conspecifics performing the same class of behavior at the same time. It has been shown, for instance, that children are more willing to taste a new food if they see an adult (32) or a peer (33, 34) eat it.

Other studies have associated food neophobia with temperamental traits, or individual differences in emotional and behavioral reactivity and regulation [e.g., refs. (35, 36)]. Differences in temperament lead to different responses to the same stimuli across individuals (37). Several temperamental traits have been found to be associated with food neophobia [for reviews, see Lafraire et al. (8) and Nicklaus and Monnery-Patris (28)]. Food neophobia is associated with higher levels of negative emotionality (38), shyness (39), lower levels of sensation-seeking (5) and approaches to novel stimuli (35). In addition, it has been shown that tactile defensiveness, overreactions to the experiences of touch or withdrawals from some typically harmless tactile stimuli (e.g., grass or sand) is related to high levels of food neophobia (40). More central to the present research, food neophobia is often connected to anxiety (11) or even disgust, over new foods (41, 42).

Recently, Maratos and Staples (43) showed that, although all children demonstrate attentional biases (e.g., facilitated visual engagement) toward new foods, these biases were heightened in children displaying higher levels of food neophobia. The three components (anxiety, disgust, and attentional biases) are standard markers of phobias (44). Moreover, high levels of neophobia are correlated with stronger typical physiological fear responses to new foods, such as galvanic skin response and an increase in pulse or respiration rhythm (45) which suggests that food neophobia is a true phobia (see ref. (46) for a review). In addition, children justify their fear by providing reasons related to the dangers of eating something they do not know (47). For instance, Johnson et al. (47) asked children between 3 and 5 years of age their reasons to avoid tasting new foods, and more than half of their justifications referred to the fear of negative consequences following ingestion (e.g., nausea, falling sick, choking, dying). An additional finding of their study is that neophobic children rated the foods less favorably than more neophilic children. Studies on non-human species also suggest that food neophobia is a real fear. For example, in rats, a lesion of the amygdala (48, 49) and infusions into this region of adrenergic agents (50, 51) are associated with a reduction in food neophobia.

Although it has been shown that food neophobia was correlated with several social and temperamental factors, there is surprisingly little research investigating whether cognitive factors could explain differences between neophobic and neophilic individuals. However, recent developmental studies point to the importance of investigating cognition as a way to further understand food-related decision-making and foster more healthy eating behaviors in children (52, 53).

At the cognitive level, recent studies uncovered a negative relationship between children’s food neophobia and category-based abilities [e.g., categorization and induction (14, 54, 55)]. For instance, in a forced-choice task, Rioux et al. (54) tested 2- to-6-year-old children’s abilities to discriminate between two taxonomic categories, vegetables and fruits. Higher levels of food neophobia predicted lower performance (see also ref. (56) for similar results). Rioux et al. (55) revealed that food neophobia and taxonomic category-based induction performance were also negatively correlated. Neophilic children tended to generalize blank properties (e.g., “contains zuline”) according to taxonomic category membership (e.g., from a green zucchini to an orange carrot) as adults generally do, whilst neophobic children tended to generalize the properties according to perceptual similarity [e.g., from a green zucchini to a green banana (55)]. Interestingly, the negative relationship between food neophobia and categorization abilities is not restricted to taxonomic knowledge but extends to thematic knowledge [e.g., the ability to associate a burger patty with a burger bun (14)]. This evidence shows a strong negative association between children’s food neophobia and their categorization performance.

However, performance is not the only indicator of participants’ behavior. The same level of performance may result from liberal or conservative strategies. For example, when asked whether some items are food or not, the accuracy of two participants can be 50%. However, one participant may have answered that all items were food (i.e., a liberal strategy), and the other that they were all nonfood (i.e., a conservative strategy).

The Signal Detection Theory [SDT (57)] separates a participant’s categorization performance and strategy into sensitivity and decision criteria respectively. The decision criteria may vary as a function of the relative costs of missing the signal (i.e., *misses*, here an opportunity to feed oneself) and responding to the noise as if it was the signal (i.e., *false alarms*, here getting poisoned). A propensity to categorize any stimulus as noise, which will result in a high proportion of misses, is described as a conservative decision criterion, whereas categorizing them as the signal, giving a high proportion of false alarms, is a liberal decision criterion.

In the food domain, Rioux et al. (54) found that food neophobia was negatively associated with sensitivity in children between 2 and 6 years of age. The authors did not observe any relationship with the decision criterion. However, they tested children’s ability to categorize vegetables and fruits, a task in which errors have no obvious costs or benefits. The task might have no effect on the decision criterion which is known to vary as a function of the perception of the risk, that is when miscategorization carries some costs [e.g., when failing to correctly identify someone as angry incurs punishment that would otherwise have been avoided (58)]. For instance, anxious individuals who have difficulties identifying facial expressions are more likely to categorize both fearful and positive emotional facial expressions as threatening than their non-anxious counterparts (59, 60). Therefore, in order to find a link between categorization strategies and food neophobia we need a task in which errors are associated with risks. A recent study by Foinant et al. (61) supports this hypothesis. The authors found that children with high levels of food neophobia had an increased likelihood of extending the negative properties from one food (such as sickness, e.g., “This food makes Feppy throw up”) to another food compared to more neophilic children, which is compatible with the hypothesis that neophobic children want to minimize the risks in the case of food. The current research tested the influence of food neophobia on children’s decision criteria in edibility judgments categorization tasks in which errors carry a risk (i.e., getting sick after eating something inedible).

As mentioned above, neophobic children have poor sensitivity in the food domain, compared to their neophilic counterparts. Decreased sensitivity makes errors more likely. We hypothesized that neophobic children mitigate this increased risk by adopting decision criteria that differ from neophilic children’s. In Experiment 1, we tested 4-to-6-year-old children who had to discriminate fruits and vegetables from nonfoods matched on color and shape [e.g., a red tomato and a red Christmas ball; see refs. (62, 63) for similar designs]. This task allowed us to measure both children’s sensitivity (i.e., categorization performance) and decision criterion (i.e., categorization strategy).

The SDT framework allows predictions on the probability of making errors as a function of perceived risk but also predictions regarding perceived uncertainty (1, 58). When a risk is involved (e.g., consuming something inedible), increased uncertainty triggers safer strategies whereas a decrease in perceived uncertainty should lead to riskier strategies (e.g., considering most of the stimuli in the environment as safe). In Experiment 2, we manipulated uncertainty through the degree of food processing, contrasting whole and sliced items. Indeed, recent studies have shown that food processing (i.e., signs of human interventions such as slicing) decreases uncertainty about edibility and is associated with food safety in adults (64–66) and children (67, 68). Manipulating the processing state of the items had two purposes. First, we tested whether children categorized differently whole and sliced items. Second, we tested whether the processing state would influence the decision criterion of neophobic and neophilic children in the same way. We formulated two opposite hypotheses. (1) Neophobic children would rely more on the cues of food processing than their neophilic counterparts who can rely on their greater accuracy. (2) Conversely, only neophilic children may rely on cues of food processing and neophobic children may display caution independently of the item states.

Based on the available literature, we expected that neophobic children would show a poorer sensitivity and a more conservative decision criterion, a tendency to say *no*, in judging items as edible or inedible compared to their neophilic counterparts. We also hypothesized a more liberal decision criterion for sliced items as compared to whole items based on the edibility cues. We expected the state of the items (i.e., whole and sliced) to reveal neophobia-related differences in categorization strategy if such differences existed. Finally, based on the above distinction between food neophobia and pickiness, we assessed whether these two conditions would differ in terms of sensitivity and decision strategies when categorizing edible and inedible substances. Differences between the two dispositions would contribute to the current debate regarding their nature and possible differences.

## Experience 1: Materials and methods

### Participants

Participants were 120 children (63 girls and 57 boys; age range = 48.20–76.20 months; mean age = 63.50; SD = 7.29). This sample size was chosen to match previous studies that found an effect of food rejection on categorization [e.g., refs. (14, 54, 61)]. They were predominantly Caucasian and came from middle-class urban areas. Informed consent was obtained from their school and their parents. The procedure was in accordance with the Declaration of Helsinki and followed institutional ethics board guidelines for research on humans.

### Materials and procedure

To measure children’s food neophobia we used the Child Food Rejection Scale [CFRS (22)]. The CFRS was developed to assess, by hetero-evaluation, 2-to-7-year-old children’s food rejection on two subscales: one is measuring children’s food neophobia and one is measuring their pickiness on a 5-point Likert-like (*Strongly disagree*, *Disagree*, *Neither agree nor disagree*, *Agree*, *Strongly agree*). Caregivers were asked to rate to what extent they agree with statements regarding their child’s neophobia (e.g., “*My child rejects a novel food before even tasting it*”) and pickiness (“*My child rejects certain foods after tasting them*”). Each answer was then numerically coded with high scores indicating higher food neophobia and pickiness (scores could range from 6 (highly neophilic) to 30 (highly neophobic) for neophobia, *M* = 14.9, SD = 5.06; from 5 (highly non-picky) to 25 for pickiness (highly picky), *M* = 16.4, SD = 4.92). We also computed a global food rejection score from 11 (highly neophilic and non-picky) to 55 (highly neophobic and picky) by adding the food neophobia and pickiness scores (*M* = 31.4, SD = 8.88). The observed range of scores is similar to the one typically found in French preschool-aged children [e.g., refs. (14, 22, 69)].

Children were tested individually for approximately 10 min in a quiet room at their school and told they will play a computer game. The experiment consisted of a familiarization phase followed by a test phase.

**FIGURE 1:**
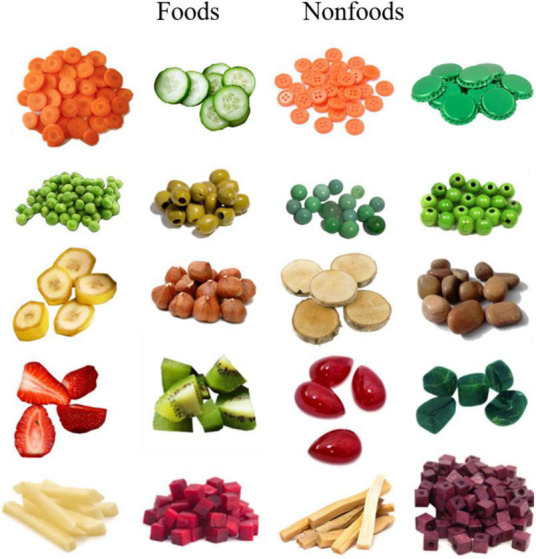
Test stimuli used in Experiment 1.

The categorization task was presented on a computer and designed with OpenSesame. Children were seated at 50 cm from a computer screen. They were instructed to respond as quickly and as accurately as possible by pressing the target button whenever a food picture appeared and by pressing the non-target button when a nonfood picture appeared. We used a real puppet named “Yoshi” in order to minimize children’s risk of transferring their own food preferences or consumption habits into the task. We adapted Rioux et al. (63) and told the children: ‘I need your help; at home, I have many things that look like foods but which sometimes are not foods. Yoshi who comes to visit me always puts anything in his mouth. But we do not want him to get sick because he ate something that was not food. Do you agree with me? Yoshi should not get hurt. Can you help me to tell him what he can eat and what he cannot eat? You press this button (pointing to the target button) when you see something that can be eaten. When you see something that cannot be eaten you press this other button (pointing to the non-target button). But be careful, Yoshi should not put things in his mouth that cannot be eaten.” The task started with a familiarization phase of eight trials (four edible plant-based foods and four nonfoods). In the familiarization phase, we explained the meaning of “things that cannot be eaten” that were real non-edible items, and that we did not refer to poisonous or unlikable (by children’ standards) foods. During the familiarization phase children also trained themselves with the response buttons and feedbacks were provided by the experimenter when they did an error. Failed trials were repeated until children succeeded. The test phase consisted of 10 target (i.e., the signal) and 10 non-target (i.e., the noise, distractors) trials presented in random order. All foods were fruits and vegetables as these two categories are the main targets of food rejection (7). Besides, the foods and nonfoods used were individually matched in color and shape (see Figure 1). For each trial, the stimulus (apparent size: 20° × 13.5°) was displayed until the child’s answer.

### Data analyses

The type of response for each food stimulus (hit or miss) and each nonfood stimulus (correct rejection or false alarm) was recorded. Each participant was assigned a hit score (i.e., number of food stimuli categorized as food), a miss score (i.e., number of food stimuli categorized as nonfood), a correct rejection score (i.e., number of nonfood stimuli categorized as nonfood), and a false alarm score (i.e., number of nonfood stimuli categorized as food). Hit, miss, correct rejection, and false alarm scores could vary between 0 and 10. These scores were used to calculate a categorization performance score, the sensitivity index A′, and a categorization strategy score, the Beta, derived from SDT (57), adapting them to experiments based on small numbers of stimuli [see ref. (70)]. SDT is used to analyze data derived from tasks where a decision is made regarding the presence or absence of a signal (i.e., the foods) embedded in noise (i.e., the perceptually similar nonfoods). The A′ represents the distance between the mean of the signal distribution and the mean of the noise distribution. The greater the A′ the better an individual is at discriminating the signal from the noise. A′ ranged from 0 to 1, with 0.5 indicating responses at chance level, and 1 maximum discriminability.

$$A^{\prime }=l\,o\,g\,\left[\frac{N_{H}+0.5}{N_{M}+0.5}\right]-l\,o\,g\,\left[\frac{N_{F\,A}+0.5}{N_{C\,R}+0.5}\right]$$

The decision criterion Beta represents the individual’s strategy to categorize stimuli as the signal rather than the noise. Beta ranged from –1 to 1, with negative values indicating a liberal strategy (i.e., children tending to categorize any stimulus as food), and positive values indicating a conservative strategy (i.e., children tending to categorize any stimulus as nonfood).

$$B\,e\,t\,a=-l\,o\,g\,\left[\frac{N_{H}+N_{F\,A}+0.5}{N_{M}+N_{C\,R}+0.5}\right]$$

N_H_, N_M_, N_FA_, and N_CR_ correspond to the numbers of hits, misses, false alarms, and correct rejections, respectively.

## Results

We assessed A′ and Beta in order to test the hypothesis that children’s categorization was impacted by their food neophobia (see Table 1).

**TABLE 1:** Descriptive statistics children’s categorization scores.

	Children (*n* = 120) *Mean* (SD)
Hit	79.8% (17.0%)
Miss	20.2% (17.0%)
Correct rejection	74.7% (16.8%)
False alarm	25.3% (16.8%)
A′	0.714 (0.120)
Beta	–0.028 (0.116)

SD, standard deviation.

Given the relatively broad age range of the children reported in this study, as shown in Table 2, preliminary Pearson’s correlations tested for significant associations between children’s age with the key variables (children’s food neophobia and pickiness scores, categorization A′ and Beta). In addition, independent *t*-tests examined differences in children’s age, food neophobia scores, food pickiness scores, and categorization scores for girls and boys. The *t*-tests did not reveal any differences between girls and boys on any of these measurements (*p* > 0.05).

**TABLE 2:** Pairwise Pearson correlation coefficients between children’s age and their A′, Beta, food neophobia, and pickiness scores.

	A′	Beta	Food neophobia scores	Food pickiness scores
Age	*r* = 0.177 *p* = 0.053 *p*_*Holm*_ = 0.372	*r* = 0.190 *p* = 0.038 *p*_*Holm*_ = 0.302	*r* = –0.148 *p* = 0.106 *p*_*Holm*_ = 0.629	*r* = –0.119 *p* = 0.196 *p*_*Holm*_ = 0.784

We performed partial Pearson’s correlations between children’s food neophobia scores and categorization scores, after controlling for age. The results revealed that food neophobia scores were significantly related to both A′ and Beta. Consistent with previous findings [e.g., ref. (54)], food neophobia was negatively associated with children’s sensitivity (A′; *r* = –0.211, *p* = 0.021). However, recall that our main question was whether neophobic (i.e., children scoring high on the food neophobia subscale) and neophilic children (i.e., children scoring low on the food neophobia subscale) would adopt the same decision criterion. Our results show that food neophobia was also positively correlated with Beta (*r* = 0.182, *p* = 0.047) which means that, as predicted, highly neophobic children adopted a conservative, protective strategy, categorizing more often actual edible substances as nonfoods and avoiding mistaking inedible substances as food compared to their more neophilic counterparts.

Although food pickiness was not correlated with A′ (*r* = –0.096, *p* = 0.297) nor Beta (*r* = –0.021, *p* = 0.824), we used the *linearhypothesis* function from the *car* package in R (71) to test the hypothesis that the difference between the regression coefficients of food neophobia and pickiness for explaining the categorization scores differed from 0. Results did not reveal a significant difference between food neophobia and pickiness to predict A′ (*t* = –1.41, *p* = 0.162). However, the results revealed that food neophobia was a stronger predictor of Beta than food pickiness (*t* = 2.33, *p* = 0.022).

## Discussion Experiment 1

In line with previous evidence [e.g., refs. (14, 54)], neophobic children in Experiment 1 performed more poorly on the categorization task than their neophilic counterparts. Our main result was that high levels of food neophobia predicted a safer categorization strategy. Indeed, neophobic children did overall more errors, even categorizing actual edible substances as nonfood. However, they also avoided dangerous errors since they categorized inedible substances as food less often than neophilic children. These results show that food neophobia was associated with a more conservative decision criterion, which was not the case for food pickiness.

## Experiment 2: Materials and methods

In the following experiment, we investigated whether food processing cues would influence children’s categorization strategies and would interact with their levels of food neophobia. According to recent evidence, food processing is a visual cue that can reduce uncertainty about edibility and thus promote feelings of safety in the food domain (61, 64–66, 68). Contrary to unprocessed food which is natural food with no signs of human intervention, processed food is defined as food that exhibits signs of human intervention (e.g., sliced). For instance, Foinant et al. (61) showed that children between 4 and 6 years generalize significantly fewer negative health properties (e.g., “makes Feppy throw up,” p. 5) to a food if it is sliced compared to whole. Here, we investigated whether children would adopt different categorization strategies for whole and sliced items and if the processed state of an item would interact with food neophobia.

### Participants

Children were recruited at their schools from the same population as in Experiment 1. None of the participants took part in the first experiment. They were 137 children (77 girls and 60 boys; age range = 57.14 to 72.07 months; mean age = 64.50; SD = 3.72). As in Experiment 1, the caregivers filled out the CFRS (food neophobia scores, *M* = 15.3, SD = 5.28; food pickiness scores, *M* = 16.8, SD = 4.41; and global food rejections scores, *M* = 32.1, SD = 8.81). As in Experiment 1, children’s CFRS scores were similar to previous studies [e.g., ref. (22)].

### Materials and procedure

**FIGURE 2:**
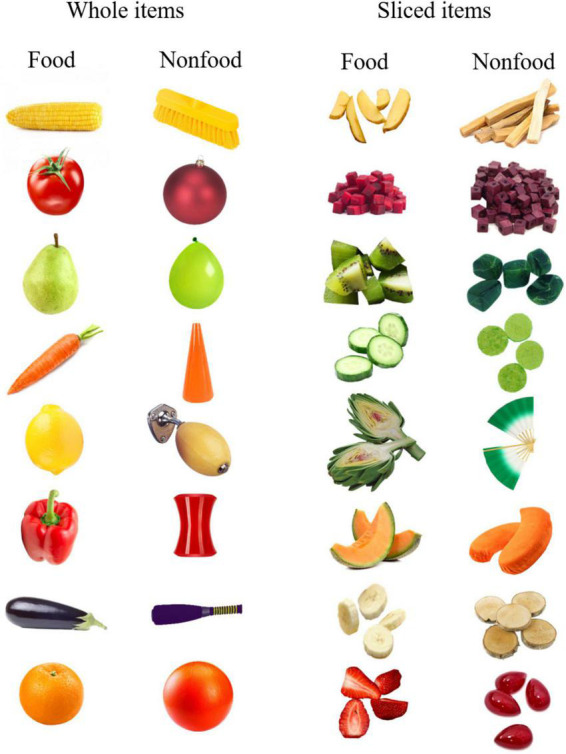
Test stimuli used in Experiment 2.

The procedure for the categorization task was the same as Experiment 1, however, we introduced the factor “item state” (whole versus sliced items) in the design. The test phase consisted of 16 target (i.e., the signal) and 16 non-target (i.e., the noise, distractors) trials presented in random order. The target trials were composed of eight whole edible food items and eight sliced edible food items. The non-target trials were composed of eight whole non-edible items and eight sliced non-edible items (see Figure 2).

## Results

We assessed A′ and Beta to test the hypothesis that children’s categorization was influenced by their level of food neophobia and item states (Whole and Sliced; results are set out in Table 3).

**TABLE 3:** Descriptive statistics for children’s categorization scores as a function of item states.

Children (*n* = 137) *Mean* (SD)	Whole items *Mean* (SD)	Sliced items *Mean* (SD)
Hit	92.6% (9.04%)	90.7% (13.1%)
Miss	7.4% (9.04%)	9.3% (13.1%)
Correct rejection	78.5% (15.8%)	57.9% (23.7%)
False alarm	21.5% (15.8%)	42.1% (23.7%)
A′	0.816 (0.113)	0.726 (0.111)
Beta	–0.081 (0.096)	–0.206 (0.194)

SD, standard deviation.

As shown in Table 4, preliminary Pearson’s correlations tested significant associations between children’s age and the study’s main variables (children’s food rejection scores and categorization scores). In addition, independent *t*-tests examined differences in children’s food rejection and categorization scores for girls and boys. The *t*-tests did not reveal any differences between girls and boys on any of these measurements (*p* > 0.05). In view of these preliminary analyses, linear mixed-effects models were used, with children serving as a random factor to account for shared variances within-subjects, controlling for age. Predictors were kept in the adjusted models following their ability to improve the model through the goodness of fit assessed using the Akaike Information Criterion [AIC (72)].

**TABLE 4:** Pairwise Pearson correlation coefficients between children’s age and their A′, Beta, food neophobia, and pickiness scores.

	A′	Beta	Food neophobia scores	Food pickiness scores
Age	*r* = –0.198 *p* = 0.037 *p*_*Holm*_ = 0.187	*r* = –0.028 *p* = 0.768 *p*_*Holm*_ = 0.768	*r* = 0.105 *p* = 0.272 *p*_*Holm*_ = 0.544	*r* = 0.236 *p* = 0.013 *p*_*Holm*_ = 0.101

### Sensitivity: A′

As shown in Table 5, the models were constructed by iteratively adding predictive variables to the null model (M0, the intercept and no predictor). Based on the procedure of decreasing the AIC (72), we constructed the model that was the best fit to the data with A′ as the outcome measure. Our best fit model (M2) contained random effects (participants), and within-subjects fixed-effects: item state (Whole or Sliced) and food neophobia (continuous factor). This model explained 15.8% of the variation across our sample, as demonstrated by the adjusted *R*^2^. We report the ANOVA output results for the model throughout.

**TABLE 5:** The goodness of fit of the linear mixed-effects models with A′ as the outcome measure.

	Model	Df	AIC	Pseudo *R*^2^	*p*
M0	1		–383.81		
M1	… + item state	1	–441.47	0.138	<0.001
**M2**	**… + item state + food neophobia**	**2**	–**444.12**	**0.158**	**0.033**
M3	… + item state * food neophobia	3	–444.55	0.162	0.122
M4	… + item state + food neophobia + food pickiness	3	–442.13	0.157	0.946

The best model is indicated in bold. M2 had the lowest AIC and, thus was the best model explaining children’ sensitivity A’ given the data.

Results revealed an effect of item state (*F* = 18.63, *p* < 0.001, *d* = 0.74) with significantly more accurate discriminations for whole (*M* = 0.816, SD = 0.113) than for sliced (*M* = 0.726, SD = 0.111) items. There was also a significant effect of food neophobia (*F* = 4.73, *p* = 0.031, *d* = –0.35). Food neophobia scores and A′ were significantly negatively correlated (*r* = –0.205 *p* = 0.017). The highly neophobic children had a lower discrimination accuracy to distinguish between food and nonfood items than the more neophilic children.

### Decision criterion: Beta

As with A′, we iteratively ran the models on children’s Beta. As shown in Table 6, the best fit model (M3) contained random effects (participants), and within-subjects fixed-effects: item state (Whole or Sliced), food neophobia (continuous factor), and the interaction item state: neophobia. This model explained 23.1% of the variation across our sample, as demonstrated by the adjusted *R*^2^.

**TABLE 6:** The goodness of fit of the linear mixed-effects models with Beta as the outcome measure.

	Model	Df	AIC	Pseudo *R*^2^	*p*
M0	1		–205.64		
M1	…+ item state	1	–257.33	0.144	<0.001
M2	… + item state + food neophobia	2	–273.44	0.211	<0.001
**M3**	… + item state * food neophobia	**3**	–**281.11**	**0.231**	**0.002**
M4	… + item state * food neophobia + food pickiness	4	–279.11	0.231	0.985

The best model is indicated in bold. M3 had the lowest AIC and, thus was the best model explaining childrenŠs decision criterion Beta given the data.

**FIGURE 3:**
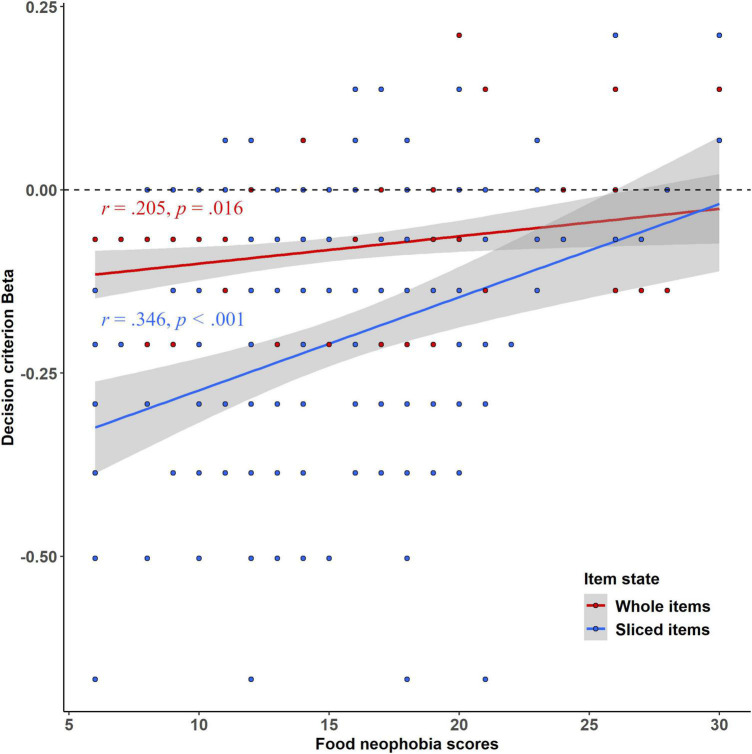
Children’s decision criterion scores Beta as a function of their food neophobia scores and item state. The Pearson coefficient correlation indicated significant and positive correlations between the children’s food neophobia scores and decision criterion Beta, for whole (*r* = 0.205, *p* = 0.016, the red line) and sliced items (*r* = 0.346, *p* < 0.001, the blue line).

Results revealed an effect of item state (*F* = 32.75, *p* < 0.001, *d* = 0.98) with significantly more sliced items categorized as food (*M* = –0.206, SD = 0.194) than whole items (*M* = –0.081, SD = 0.39), indicating that children were more willing to decide that a sliced item was a food rather than a whole item. There was also a significant effect of food neophobia (*F* = 19.36, *p* < 0.001, *d* = 0.20), with highly neophobic children categorizing fewer items as foods than other children, thus being more conservative. Food neophobia scores and Beta were significantly positively correlated (*r* = 0.354, *p* < 0.001). Figure 3 shows a significant interaction between item states and food neophobia scores (*F* = 10.02, *p* = 0.002, *d* = 0.54). Food neophobia scores were more strongly positively correlated with Beta for sliced items (*r* = 0.346, *p* < 0.001, blue line in Figure 3) than for whole items (*r* = 0.205, *p* = 0.016, red line in Figure 3). The neophilic children were more liberal for sliced items than their neophobic counterparts, categorizing more often the sliced items as foods. On the other hand, neophobic children treated whole and sliced items similarly, adopting a more conservative strategy than their more neophilic counterparts.

## Discussion experiment 2

Experiment 2 built upon the findings from Experiment 1 and assessed the effect of the state of the stimuli with whole and sliced items. We found that higher levels of food neophobia were predictive of poorer sensitivity and a more conservative strategy. An important additional finding of this second experiment is that neophilic children were more liberal for sliced stimuli than for whole stimuli. Neophobic children, on the other hand, were conservative, independently of the item states. Finally, as in the first experiment, only food neophobia has been retained in the models. No significant effect was obtained regarding food pickiness.

## General discussion

The present research aimed to assess the contributions of performance and strategy in the categorization of edible and inedible items by neophobic and neophilic children. In two experiments, we used a discrimination paradigm within the framework of SDT.

In line with previous studies (14, 54–56, 63) neophobic children in the present research performed poorly in the categorization tasks compared to their neophilic counterparts. Our main contribution is that food neophobia affected children’s taxonomic categorization ability of both edible and inedible items, hence not only food categories (e.g., vegetables and fruits). Taken together, the evidence suggests that neophilic children have better discrimination abilities and are, therefore, expected to be protected from trying to consume inedible substances, whereas neophobic children are expected to be at risk of consuming them. However, although neophilic children in our experiments were better at discriminating food from nonfood items, their strategies put them at a higher risk of accepting nonfoods as edible than neophobic children.

Previous studies on neophobia within the SDT framework did not reveal any difference in categorization strategies between neophobic and neophilic children [e.g., ref. (54)]. In the present experiments, neophobic children favored increased misses whereas neophilic children had a higher rate of false alarms, categorizing more nonfood items as edible. Because neophobic children cannot accurately identify foods from nonfoods, they may compensate with more conservative strategies to avoid errors. Paradoxically, neophilic children, who were more accurate at discriminating foods from nonfoods, adopted a more liberal and riskier strategy. This is adapted in most daily situations in which food safety is the norm. Indeed, in our contemporary food environment and modern societies, food safety is controlled in food supply chains, and conservative strategies are less useful. Experiment 2 is in line with this interpretation. We expected that combining perceptual similarity between foods and nonfood items with signs of food processing would increase the number of edibility judgments for sliced items compared to whole items. Interestingly, the item state did not affect neophobic children’s categorization, whereas neophilic children adopted a more liberal strategy for sliced items than for whole items. In other words, only the neophilic children detected or used the safety cues conveyed by food processing (64–68).

Together, the neophobic lower performance in discriminating foods from nonfoods is consistent with their strategy to report more items as inedible, including processed foods, suggesting lower levels of confidence. The present findings illuminate the fact that neophobic children seem less impacted by interventions that aim to overcome food rejection (56, 73, 74) because they experience such eating situations as more threatening than other children.

Last, it is worth mentioning that only the neophobia dispositions significantly correlated with children’s categorization and were kept in our models. We obtained no significant effect of food pickiness [contrary to ref. (54)]. This contrast between food neophobia and pickiness was also found in several previous studies [e.g., refs. (61, 63)]. For instance, Foinant et al. (61) witnessed that only food neophobia but not food pickiness was predictive of an increased likelihood of generalizing negative properties of a food to other foods. These results suggest that these two dispositions do not have the same influence on children’s decisions about food. From a theoretical standpoint, it seems more compelling that food neophobia, rather than food pickiness, has a more robust link with increased conservative decision criteria. Indeed, reviews on food neophobia postulate that food neophobia is considered to be an adaptive mechanism that promotes survival (1, 30, 75). Furthermore, as mentioned in the introduction, food neophobia increases feelings of anxiety and physiological response, an outcome not evidenced in food pickiness (11).

Several limitations of this research need to be addressed. First, our food stimuli were only made of fruits and vegetables, which are the main targets of food neophobia (7). However, it would be interesting to investigate children’s categorization abilities to discriminate between foods and nonfoods with categories that are less prone to neophobia (such as starchy food). Second, we equated food processing with slicing. Evidence suggests that food processing is a matter of degree (65). For instance, other processing techniques modifying organoleptic properties of foods, such as cooking, could affect edibility judgment not only in neophilic children but also in neophobic children. Indeed, current evidence regarding the interaction of food neophobia and food processing is scarce and, possibly, neophobic children may need stronger safety cues to overcome their fear about a potential food source. Similarly, morphing techniques (e.g., to create stimuli on finely graded continua ranging, for instance, from an edible unfamiliar food of red color to an inedible, even poisonous, unfamiliar food in green) would allow performing analyses at various points along the continuum of threat intensity. Third, food neophobia is not the only individual characteristic that can influence food categorization. In adults, previous work has shown that hunger level, dietary habits, and BMI could also explain differences in food categorization [e.g., ref. (76)]. It might be informative to measure these individual characteristics alongside children’s food neophobia. Finally, we used a puppet procedure to decrease the risk of children using their preferences and consumption habits to answer the task. This procedure is widely used in many categorization and generalization tasks, and far beyond, in the cognitive development literature. Although some researchers have questioned the validity of using puppets [e.g., ref. (77)], they have not yet been backed by empirical evidence. Instead, studies that assessed the use of puppets in research on young children’s cognitive development found that “it makes no difference if the protagonist is presented as a real person, a puppet, a doll, a pictured storybook character, or a videotaped person” [p. 664, ref. (78), regarding false belief understanding; see also Li et al. (79) regarding knowledge learning]. Nevertheless, future studies could consider comparing the impact of people and puppets on children’s edibility judgments.

Despite these limitations, the current research has strong implications for theories of food neophobia. Food neophobia may shape children’s strategies that may reinforce the rejection of novel but perfectly safe foods. As neophobic children engage in risk-avoidant decision-making, consequent behavioral avoidance may prevent children from gaining experience and knowledge in the food domain, thereby eliciting a self-perpetuating cycle. If children exhibit conservative strategies, caregivers may be discouraged from exposing them to new foods and eating situations. Consequently, the learning opportunities of foods and eating situations may be greatly reduced, maintaining poor knowledge about food and the conservative strategy compensating it. The current experiments provide only indirect evidence for this cycle. Further research is needed to examine the possibility that risk-avoidant decision-making serves to reinforce pre-existing individual differences in neophobia.

Current studies also have implications for theories of neophilia. While we worry that neophobic children will reject new foods that are important for healthy development, in the present research, neophilic children dangerously accepted as edible nonfood items. It is currently believed that the number of accidental poisoning among young children is due to difficulties in making the distinction between food and nonfood items (80). However, our data suggest that the attraction toward trying inedible substances may, instead, reflect a dangerously liberal decision criterion.

Finally, the current findings open up new perspectives for practical interventions to promote healthy eating. Current interventions aiming at fostering dietary variety tend to deploy the same program equally to all children of the same age group. However, our data strongly underline the crucial importance to take into account the individual factors that may modulate the extent to which children may benefit from such interventions.

## Data availability statement

The raw data supporting the conclusions of this article will be made available by the authors, without undue reservation.

## Ethics statement

The studies involving human participants were reviewed and approved by official agreement between the Academia Inspection of Côte d’Or and the University. Written informed consent to participate in this study was provided by the participants’ legal guardian/next of kin.

## Author contributions

DF, JL, and J-PT conceived the hypotheses and the design of the study. DF collected the data and performed the statistical analyses. All authors contributed to the manuscript writing, read, and approved the submitted version.
